# Conservative management for iatrogenic gastric perforation by transesophageal echocardiography

**DOI:** 10.1186/s40981-018-0189-7

**Published:** 2018-06-26

**Authors:** Masaya Oshiro, Hirotsugu Kanda, Akane Oshiro, Kenta Kure, Megumi Kanao-Kanda, Hiroyuki Kamiya, Takayuki Kunisawa

**Affiliations:** 1 Department of Anesthesiology and Critical Care Medicine, Asahikawa Medical University, Midorigaoka-higashi 2-1-1-1, Asahikawa, Hokkaido 078-8510 Japan; 2 Department of Cardiac Surgery, Asahikawa Medical University, Midorigaoka-higashi 2-1-1-1, Asahikawa, Hokkaido 078-8510 Japan

**Keywords:** TEE, Complication, Gastric perforation

## Abstract

**Background:**

Though several cases of upper gastrointestinal tract injury caused by transesophageal echocardiography (TEE) have been reported, gastric perforation is very rare. Herein, we report the case of TEE-associated gastric perforation that was successfully treated conservatively.

**Case presentation:**

An 82-year-old man underwent mitral valve repair. Postoperative esophagogastroduodenoscopy and computed tomography revealed gastric perforation. Surgical treatment was initially considered, but conservative management was selected to avoid increasing operative stress, to minimize the need for total gastrectomy (including the lower esophagus), and to minimize the risk of a potential intraperitoneal infection spreading to the thoracic cavity.

**Conclusion:**

Conservative management of gastric perforation can be successful even when the perforation is recognized later than 12 h following the event, provided that there are no abdominal symptoms and no signs of peritoneal effusion or sepsis. Our experience suggests that conservative management is a feasible option for treating TEE-associated gastric perforation in appropriately selected cases.

## Background

Transesophageal echocardiography (TEE) is widely used for perioperative hemodynamic monitoring in clinical settings [1]. However, numerous complications including oropharyngeal, laryngeal, esophageal, and gastric injury have been reported [2], as TEE is a semi-invasive monitoring modality, unlike transthoracic echocardiography. While several reports have described TEE-associated upper gastrointestinal tract injuries including bleeding, erosion, laceration, and perforation, gastric perforation represents a very rare complication [2–5]. Herein, we report a case of gastric perforation by TEE that was treated conservatively, without surgical procedures.

## Case presentation

An 82-year-old man (height, 167 cm; weight, 58 kg) was scheduled for mitral valve repair due to exacerbated heart failure symptoms. His past medical history included aortic valvular stenosis with post-aortic mechanical valve replacement and atrial fibrillation treated with oral warfarin. The patient had no upper gastrointestinal symptoms or related medical history. Written informed consent was obtained from the patient.

Four days before mitral repair surgery, warfarin was replaced with intravenous heparin (10,000 units/day), which was administered until 6 h preoperatively. Under general anesthesia, a TEE probe was inserted, without resistance, to evaluate perioperative cardiac function and residual regurgitation using mid-esophageal and trans-gastric views, as appropriate. The inferior vena cava (IVC) could not be visualized in trans-gastric view, despite several manipulations of the probe with the venous cannula placed into the IVC. After cardiopulmonary bypass (CPB), an unexpectedly large echo-free space in the stomach was detected on TEE. The mitral valve repair was successfully undergone, the TEE probe was removed, and a nasogastric tube was inserted. Surprisingly, 700 mL of dark brown blood was aspirated from the nasogastric tube. We suspected upper gastrointestinal injury, but the patient was transferred to the intensive care unit, as the blood was dark brown and the bleeding appeared to have stopped.

**Fig. 1:**
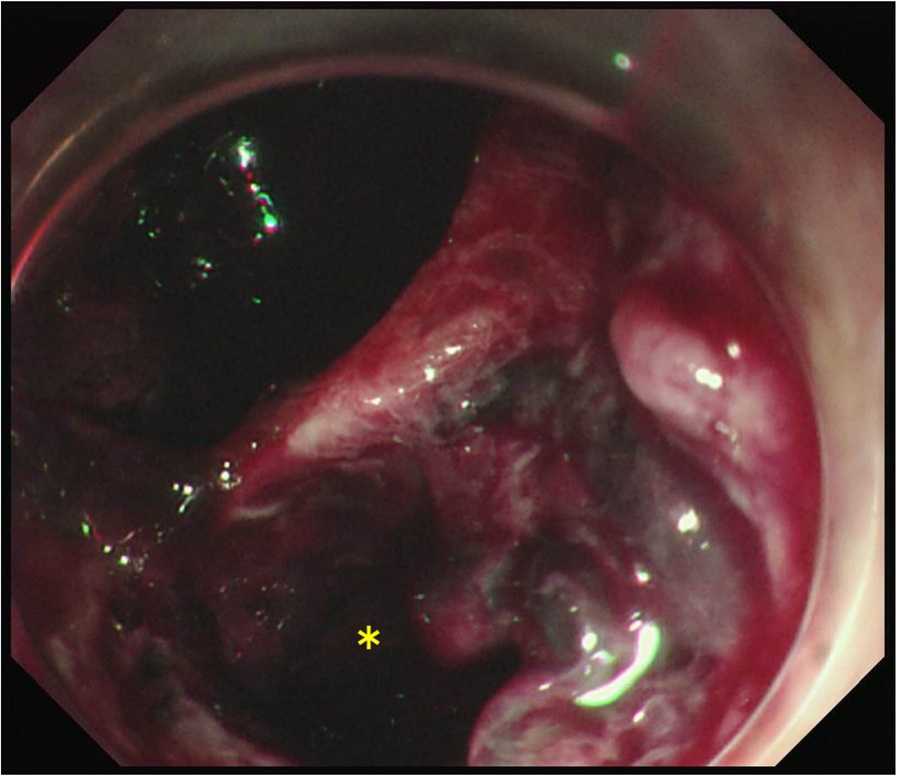
Esophagogastroduodenoscopy (EGD) image on postoperative day 1, showing a mucosal laceration spanning from the esophagogastric junction to the gastric body, with a perforation in the center of the laceration (asterisk). The EGD probe could be easily passed through the perforation site

**Fig. 2:**
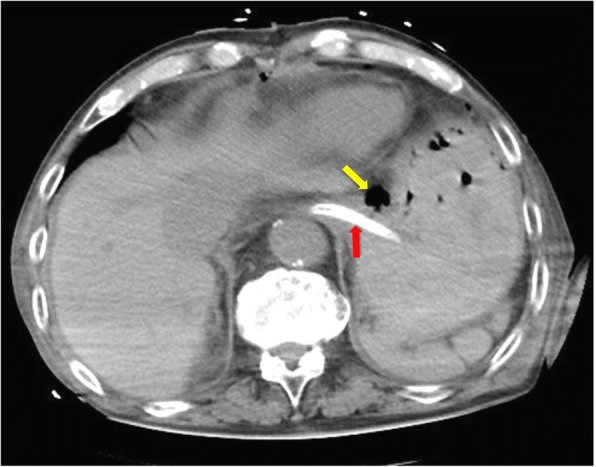
Computed tomography image on postoperative day 1, showing localized free air in the abdominal cavity. The yellow arrow indicates an area of free air between the left lateral segment of the liver and the lesser curvature of the stomach. The red arrow indicates the nasogastric tube from the lower esophagus to the cardia

Approximately 6 h postoperatively, another 100 mL of fresh blood was aspirated from the nasogastric tube. Thus, we immediately performed an esophagogastroduodenoscopy (EGD) examination and computed tomography (CT) scan. The EGD examination showed a mucosal laceration spanning from the esophagogastric junction to the gastric body, with a perforation in the center of the laceration (Fig. 1). On this occasion, a clip was placed to stop the hemorrhage from the injured area, but the perforation was too large to close with the clip under EGD. The CT scan showed localized free air in the abdominal cavity, which helped establish a definite diagnosis of gastric perforation (Fig. 2). Initially, surgical treatment was considered for the repair of the perforation. However, we selected conservative management to avoid increasing operative stress, to minimize the need for total gastrectomy (including the lower esophagus), and to reduce the risk of infection spread. Fortunately, spontaneous closure was expected, as there were no abdominal symptoms and the perforation site was covered with the lesser omentum and inferior surface of the liver.

**Fig. 3:**
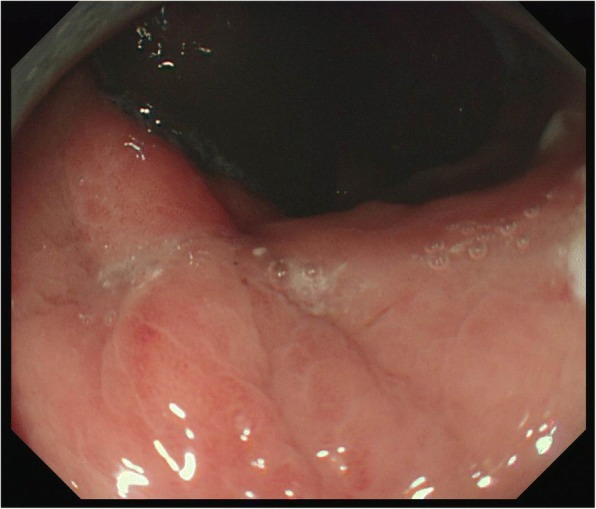
Esophagogastroduodenoscopy image on postoperative day 23, showing the perforation site completely healed, with scarring

Nasogastric tube aspiration was continued postoperatively, and the patient was administered total parenteral nutrition. On the day of surgery, the patient received hemostatic therapy with carbazochrome sodium sulfonate hydrate (100 mg) and tranexamic acid (0.5 g), as well as routine antibiotics including sulbactam sodium (0.5 g) and ampicillin sodium (1.0 g). Tazobactam sodium (0.5 g) and piperacillin sodium (4.0 g) were initiated after diagnosis of gastric perforation and continued until the perforation site had healed. Intravenous omeprazole sodium (20 mg) was used as a proton pump inhibitor until oral intake. On postoperative day 1, hemostatic therapy included carbazochrome sodium sulfonate hydrate (200 mg), tranexamic acid (1.5 g), and menatetrenone (20 mg). As there were no persistent abdominal symptoms and the nasogastric aspirate contained no blood, the tracheal tube was removed on postoperative day 3. A follow-up CT scan revealed a reduction in the echo-free space in the abdominal cavity, and upper gastrointestinal examination revealed no leaking of the contrast medium into the abdominal cavity. Thus, the patient began enteral nutrition on postoperative day 10 and oral intake on postoperative day 12. To prevent rebleeding, anticoagulation was restarted only after enteral nutrition was established (warfarin, 3 mg). To protect the gastric mucosa, oral esomeprazole (20 mg) and ecabet sodium (2000 mg) were started upon establishment of oral intake and continued until after discharge. A follow-up EGD examination on postoperative day 23 revealed that the perforation site had healed, with scarring (Fig. 3). The patient recovered without any complications and was discharged from the hospital on postoperative day 24.

## Discussion

The present report described the successful conservative management of TEE-associated gastric perforation. The unexpectedly large echo-free space and continuous bleeding from the nasogastric tube were indicative of perforation. EGD and CT were useful imaging modalities for establishing a definite diagnosis. We selected conservative management to avoid highly invasive procedures and minimize the risk of a potential intraperitoneal infection spreading to the mediastinum, implanted mechanical valve, and mitral ring. Furthermore, the patient presented no typical abdominal symptoms including pain, muscular guarding, or Blumberg’s sign, and the perforation site was covered by the lesser omentum and inferior surface of the liver. Conservative management was considered successful, as the patient had recovered sufficiently to resume oral intake by postoperative day 12.

TEE is widely used in cardiac surgery. Although TEE is considered minimally invasive, some complications have been reported. The overall incidence of TEE-associated morbidity and mortality is 0.2 and 0%, respectively [2]. The most common complication is severe odynophagia (0.1%), followed by dental injuries (0.03%), endotracheal tube malpositioning (0.03%), upper gastrointestinal hemorrhage (0.03%), and esophageal perforation (0.01%) [2]. In addition, one report described splenic injury caused by the TEE probe [6]. While upper gastrointestinal perforation is an unusual complication in TEE, it can often become severe. In the present case, the perforation may have been caused by the TEE probe being advanced and withdrawn repeatedly to maintain an anteflexed position in the stomach, allowing visualization of the venous cannula in the IVC. In order to prevent such complications, it is necessary to select the appropriate TEE probe size, as well as to insert and operate the TEE probe gently.

Min et al. reported that the risk factors for TEE-associated perforation were advanced age, prolonged CPB or ventilatory support, intracardiac procedures, post-CPB hypotension, low cardiac output, use of vasopressors or an intra-aortic balloon pump, emergency operations, and systemic anticoagulation or aspirin therapy [7]. Several of these risk factors were present in the case reported here, including advanced age, prolonged CPB (154 min), and anticoagulation therapy (warfarin).

Many reports have described upper TEE-associated gastrointestinal perforation [8–11]. Here, we report on TEE-related perforation of the stomach. Gastric perforation is a clinical condition different from esophageal perforation. Several cases involving esophageal perforation by TEE were reported, including a few cases with successful conservative management of the perforation [8, 9]. On the other hand, gastric perforation is a very rare complication in TEE, with only three such cases being reported, all treated via surgical intervention [10, 11]. Therefore, to the best of our knowledge, the present case report is the first to describe the successful conservative management of iatrogenic gastric perforation by TEE. In previously reported cases, open or laparoscopic surgery was performed for iatrogenic gastric or esophageal perforation by TEE [10, 11], in an effort to prevent severe infection-related complications such as mediastinitis, peritonitis, and septic shock, which may occur when treatment for upper gastrointestinal perforation by TEE is delayed.

The surgical management of gastrointestinal tract perforations is now often replaced by conservative therapy [12]. For example, Merchea et al. reported that non-operative management of EGD-associated upper gastrointestinal perforation can be successful when there is no evidence of contrast extravasation or free fluid on radiographic studies [13]. According to the European Society of Gastrointestinal Endoscopy Position Statement, conservative management of iatrogenic gastric perforation by EGD is recommended as a first-line treatment when the following criteria are satisfied: late recognition of perforation (> 12 h), asymptomatic presentation, and no signs of peritoneal effusion or sepsis [14]. Clipping under EGD or surgery may also be recommended in some cases of EGD-associated gastric perforation, depending on clinical symptoms or the size of the perforation site [14]. The incidence of perforation is very similar between TEE and EGD examinations [2, 7, 8, 14], as such examinations have much in common, including the size of the probe and the period of fasting. Therefore, as with EGD-associated perforation, TEE-associated gastric perforation may also be amenable to conservative management. In the present case, asymptomatic iatrogenic perforation was diagnosed at > 12 h following mitral valve repair. Moreover, open abdominal surgery was considered too invasive and might have allowed a potential intraperitoneal infection to spread to the implanted mechanical valve. Thus, we decided to perform conservative management in an effort to reduce the risk of severe complications. Had the patient presented abdominal symptoms of peritonitis (abdominal pain, Blumberg’s sign, muscular defense, fever, tachycardia), an invasive procedure to repair the perforation would have been required, per the current guidelines [14].

In conclusion, based on our experience with the patient described in this report, iatrogenic TEE-associated gastric perforation, which is a very rare complication of TEE, can be successfully managed conservatively even when the perforation is recognized later than 12 h following the event, provided that there are no abdominal symptoms and no signs of peritoneal effusion or sepsis.

## Abbreviations

CPB: Cardiopulmonary bypass
CT: Computed tomography
EGD: Esophagogastroduodenoscopy
IVC: Inferior vena cava
TEE: Transesophageal echocardiography
